# False Belief Understanding in Deaf Children With Cochlear Implants

**DOI:** 10.1093/deafed/enab015

**Published:** 2021-06-28

**Authors:** Agnieszka Pluta, Magdalena Krysztofiak, Małgorzata Zgoda, Joanna Wysocka, Karolina Golec, Joanna Wójcik, Elżbieta Włodarczyk, Maciej Haman

**Affiliations:** Faculty of Psychology, University of Warsaw, Warsaw, Poland; Institute of Physiology and Pathology of Hearing, World Hearing Center, Warsaw, Poland; Faculty of Psychology, University of Warsaw, Warsaw, Poland; Institute of Physiology and Pathology of Hearing, World Hearing Center, Warsaw, Poland; Faculty of Psychology, University of Warsaw, Warsaw, Poland; Faculty of Psychology, University of Warsaw, Warsaw, Poland; Institute of Physiology and Pathology of Hearing, World Hearing Center, Warsaw, Poland; Institute of Physiology and Pathology of Hearing, World Hearing Center, Warsaw, Poland; Faculty of Psychology, University of Warsaw, Warsaw, Poland

## Abstract

Theory of mind (ToM) is crucial for social interactions. Previous research has indicated that deaf and hard-of-hearing children born into hearing families (DoH) are at risk of delayed ToM development. However, it is unclear whether this is the case for DoH children who receive cochlear implants (CIs) before and around the second year of life. The present study aimed to investigate false belief understanding (FBU) in DoH children with CIs. The relationships between false belief task (FBT) performance, sentence comprehension, age at implantation, duration of CI use, and Speech Recognition Threshold were explored. A total of 94 children with typical levels of hearing (TH) and 45 DoH children (age range: 3–8), who received their first CI between 6 and 27 months of age, were tested on the FBT and a sentence comprehension test. Results showed that 4- and 5-year-old children with CIs performed significantly worse than their peers with TH on the FBT; 6- to 8-year-old children with CIs performed similarly to age-matched children with TH. Age at implantation and duration of CI use were correlated with sentence comprehension but not with the FBT. The results indicated that FBU was delayed until the age of 6 years in most of children with CIs.

## Introduction

Theory of mind (ToM) is the capacity to represent the internal, unobservable mental states (beliefs, desires, emotions) of other people, to infer them from situational cues, and to use them to predict others’ behavior. Such abilities are absolutely essential for effective social interaction and communication. It has been demonstrated that performance on ToM tasks correlates with social competencies, popularity among peers (Slaughter et al., 2015), leadership (Peterson et al., 2016), and (negatively) with loneliness (Devine & Hughes, 2013).

Early studies of ToM development postulated a gradual construction of these abilities, with passing false belief tasks (FBTs) being the most important milestone in this process (see Wellman, 2017). The unexpected transfer task (often called the “Sally–Anne test”) is one of the most common FBTs (Bloom & German, 2000). In it, participants must identify that a protagonist holds a false belief about the location of an object by understanding that the person lacks knowledge that the object has been moved. The participant is then asked to efficiently predict the actions of the protagonist (Wimmer & Perner, 1983). As first shown by Wimmer and Perner (1983), and subsequently replicated in a myriad of studies, this ability progresses in typically developing children according to an age-related pattern. A major change in FBT performance occurs between the ages of 3 and 5 years (Blijd-Hoogewys & van Geert, 2017; Liu et al., 2008; Perner & Roessler, 2012; for a meta-analysis on false beliefs, see Wellman et al., 2001). Previous studies have shown that typically developing 3-year-olds fail FBTs, while almost half of 4-year-olds and a majority of 5-year-olds are able to appreciate that the character holds a false belief and provide a correct answer (Wellman et al., 2001; Wysocka et al., 2020). Beyond this age, children begin to understand second-order false beliefs (“A thinks that B thinks X”; Beaudoin et al., 2020).

Converging empirical evidence strongly supports the role of language and conversational experiences in the typical development of explicit false belief understanding (FBU) (de Villiers & de Villiers, 2014; Farrar et al., 2017; Lohmann & Tomasello, 2003; Milligan et al., 2007; Peterson, 2020; Ruffman et al., 2002). Therefore, children with atypical language acquisition may be at risk of later ToM development.

One population which may be of interest in this respect is children who are deaf or hard-of-hearing (DHH). Approximately 95% of such children are born to families with typical levels of hearing (TH) (Mitchell & Karchmer, 2004). Due to the challenges that can arise in making spoken language accessible, children who are DHH are usually not immersed in a language-rich environment until interventions, such as hearing aids or cochlear implants (CIs), are implemented and hearing habilitation is provided.

The introduction of hearing screening programs for newborns has led to earlier identification of childhood hearing loss and the use of interventions (Mueller-Malesińska et al., 2000; Skarzynski et al., 2015), including cochlear implantation, which can now be performed in children under 12 months of age (Holcomb & Smeal, 2020). Although a CI does not bring hearing to a typical levels, a number of studies have reported effects of cochlear implantation on oral language development (Anderson et al., 2005; Niparko et al., 2010; Paluch et al., 2019; Ruben, 2018; Szuchnik et al., 2001; Zgoda et al., 2019), literacy outcomes (see the review by Mayer & Trezek, 2018), as well as a link with improved communication and social competence (Leigh et al., 2009). Specifically, children with CIs have demonstrated progress in early-developing language skills such as speech identification, receptive and expressive vocabulary, sentence comprehension (Duchesne & Marschark, 2019), as well as in more complex and later developing skills such as narrative abilities (Boons et al., 2013) or reading comprehension (Mayer & Trezek, 2018).

Nevertheless, CIs may not be sufficient to overcome slower development in several aspects of spoken language (Duchesne & Marschark, 2019). For example, it has been reported that children with CIs experience delays in various domains of language development, including grammar and pragmatic skills (Rinaldi et al., 2013) and lexical comprehension (Caselli et al., 2012). It has also been demonstrated that deaf children of hearing parents (DoH), including children with CIs, have less exposure to “mentalistic” conversation (including the sharing of thoughts or feelings) because their caregivers use mental-state talk less frequently than hearing parents of children with TH (Moeller & Schick, 2006; Morgan et al., 2014). This finding might be related to the results of a study by Hoff-Ginsberg (1994) that found that mothers tend to talk more to children with greater language abilities. Therefore, it is plausible that because the receptive and expressive skills of children who are DoH might be below their chronological age in early childhood (although the difference in these skills between children with CIs and children with TH gradually closes; Wie et al., 2020), they may elicit lower quality talk from their parents, specifically, with less use of mental state terms such as “believe”, “think”, or “know”. Thus, they may have fewer opportunities for incidental learning of such concepts in their immediate social environment.

All in all, two fundamental building blocks for typical ToM development—development of language (spoken or signed) and access to mind-related conversational input—could be affected in early childhood in DoH who are CI users (Peterson, 2020). Therefore, even though implantation is typically now performed by 12 months of age, most often bilaterally (Varadarajan et al., 2021), children who are DoH may be still at risk of slower ToM development than their peers with TH.

Studies on prelingually DoH children who use CIs provide inconsistent results regarding ToM competencies. For example, Ketelaar et al. (2012) showed that children aged 2–4 with CIs are skilled in early ToM concepts (understanding desire and intentions), but they do not have false belief (FB) understanding, despite having the same level of language skills (as measured by the Language Comprehension Scales from the Child Development Inventory) as controls with TH. In contrast, Remmel and Peters (2009) showed little to no differences in ToM development in children with CIs relative to their peers with TH. However, they recruited children with a relatively wide age range (3–12 years) and varied age at implantation (1.1–6.0 years). Therefore, this study does not provide direct results about the developmental trajectory of ToM during the transition between 3 to 5 years of age. It is possible that the performance of older children biased the results. Ziv et al. (2013) reported no differences between a group of 20 children with CIs using spoken language as their main mode of communication (mean age = 6.6 years; mean age at implantation = 2.5 years) and 23 peers with TH in either affective perspective-taking or in change-of-location FBU. Although the study revealed a relatively high average success rate of children with CIs on tasks measuring different domains of social development, the authors highlighted greater heterogeneity in ToM performance among children with CIs than in children with TH. This means that some children with CIs may still not have the same ToM development as their peers.

Meristo et al. (2016) demonstrated that children with CIs did not differ from their peers with typical levels of hearing on the verbal FBT (elicited-response task). However, they failed the implicit FBT, meaning that they were not able to spontaneously anticipate another person’s behavior based on their belief. This is a surprising result, as the majority of studies on children with TH show that implicit FB understanding, often referred to as the tendency to automatically take others’ beliefs into account, develops before the ability to purposely reason about others’ mental states—referred to as explicit ToM (Low & Perner, 2012). Most nonverbal FBTs measure either the participants’ gaze in anticipation of the protagonist’s belief-based behavior or the duration of gaze fixation as a reaction to an unexpected outcome, such as the protagonist behaving incongruently with their belief, both being regarded as indicators of mentalizing (Onishi & Baillargeon, 2005; Southgate et al., 2007). This is in line with an earlier study by Meristo et al. (2012) on implicit ToM in children with TH and infants who are DoH. The “DoH” infants performed worse than their peers with typical levels of hearing at anticipatory looking ToM tasks. The aforementioned results regarding implicit ToM performance in children who are DoH suggest that their ability to spontaneously attribute mental states to others might be delayed, which, according to the authors, might be explained by the lower quality of the conversational environment between children who are DoH and their parents.

Sundqvist et al. (2014) tested eight children with implantation <27 months of age and eight children with implantation >27 months of age, all aged between 4.25 and 9.5 years. Both groups lagged behind their peers with TH in their understanding of the concept of false beliefs. However, children who received their implant before 27 months of age performed the same as children with TH on a social–emotional ToM test that requires understanding someone else’s emotional states. This study demonstrates that earlier implantation might foster social development in children who are DHH by providing (via auditory stimulation) the opportunity to experience more verbal interactions with their hearing caregivers. This is in line with the novel longitudinal study of Yu and collaborators (Yu et al., 2021), which showed that children who are DoH with more advanced language abilities had better ToM growth. It is an important study on ToM development in DHH because, in contrast to previous studies, the authors examined a sizable sample (*n* = 84) of young DoH children (ages 3–6) with relatively early use of CIs or hearing aids (mean age at provision of hearing aids or cochlear implants = 1.7 years). The results yielded the typical sequential progression of performance on ToM scales found in other studies with children with TH (for example, children successfully judge what others do or do not know before they make successful judgments about others’ false beliefs; Wellman et al., 2011). However, only 3% of children who are DHH succeeded in the FBT in the first measurement. The success rate increased to 8% 6 months later, but it was still dramatically lower than in studies on children with typical hearing, which show that the majority of 5-year-olds pass FBTs (Wellman et al., 2001).

This early discrepancy in FBU might also persist in adolescents with CIs, as reported by Figueroa et al. (2020). This means that adolescents with CIs might experience problems with inferring second- or higher order beliefs and understanding multiple perspectives within a communicative situation (also referred to as mature/advanced ToM).

The results of the most recent studies are mixed. This might partly result from the fact that the protocol for when a child should receive a CI has changed quite recently. Therefore, even in relatively recent studies (Sundqvist et al., 2014), participants varied substantially in terms of age at implantation. Consequently, the age range of the participants recruited to these studies was also wide and, in most cases, children were recruited at an age when they should have had FBU years ago. Therefore, the fact that children with CIs did not differ from children with TH in ToM performance did not rule out the possibility that they had acquired the concepts necessary for understanding false beliefs later than their peers with TH.

Moreover, participants varied considerably in terms of communication modality options chosen by families (ranging from exclusively auditory, through bilingual/bicultural to exclusively visual). In a number of studies (see for example, Peterson et al., 2016; Yu et al., 2021), children with CIs used both spoken language as well as sign language, which makes it possible that their early communication experiences were qualitatively different from those of children who used only one mode of communication.

Also, there are a limited number of tasks that make minimal demands on language and working memory while examining understanding of mental states. A number of authors use short narratives accompanied by illustrations or puppet play to evaluate ToM competence (see Ketelaar et al., 2012; Sundqvist et al., 2014). Because the linguistic complexity of ToM tasks might mask children’s ability to attribute mental states to others by drawing their attention away from the other person’s perspective, even in populations with typical levels of hearing (Rubio-Fernández & Geurts, 2013; Wellman et al., 2001), it is highly plausible that the linguistic complexity of ToM tasks further hampers the performance of children with CIs on FBTs. Therefore, further investigations must be conducted on ToM in children with CIs with the use of ToM tasks that minimize executive and linguistic demands.

Taken together, these results suggest that further research is needed to document whether decreasing the age of pediatric cochlear implantation allows children to achieve typical ToM skills. Novel studies should also apply protocols with minimized language load to effectively disentangle which problems with FB understanding observed in DoH with CIs result from difficulties with the attribution of mental states and which are due to the linguistic load of these tasks.

Therefore, the following questions were investigated in the present study:

1) Do children who have used CIs from at least 27 months of age differ in their capacity for belief attribution when compared with peers with TH?
2) What is the relationship between belief attribution, sentence comprehension, age at implantation, and duration of CI use?

We examined ToM with a novel computerized task that enabled us to address the limitations of the previous studies and reduce the executive and linguistic demands.

Based on the reported effects of CIs on language acquisition, especially if provided early in life, we expected that the development of FBU would be less protracted than reported in prior ToM studies on children who are DHH. However, given that children with CIs may still experience challenges with oral language development (Colletti, 2009), we expected that the performance of children with CIs would be lower on false belief and sentence comprehension tasks than their peers with typical hearing.

## Methods

### Participants

The study sample consisted of two groups: children with CIs and children with TH. Children with CIs were recruited in clinics by an educator of the DHH. Children with TH were recruited from three local kindergartens. Written informed consent was obtained from the parents/caregivers of the children participating in the study. All children assented to taking part in the experiment. Recruitment and experimental protocols were approved by the Ethics Committee of the University of Warsaw and were conducted in accordance with the World Medical Association’s Declaration of Helsinki.

Cochlear implant users (CI group): 45 children with profound deafness (hearing loss over 90 dB in their better ear) who used CIs participated in the study (age range: 3.25–8.25 years, *M* = 5.38, *SD* = 1.15; 3-year-old: *n* = 2, 4-year-old: *n* = 10, 5-year-old: *n* = 9; 6-year-old: *n* = 18; 7-year-old: *n* = 5; 8-year-old: *n* = 1; boys: *n* = 19, girls: *n* = 26). All these children had onset of prelingual, sensorineural hearing loss. They had no diagnosis of developmental, systemic, genetic, or metabolic disorders, or major medical complications during pregnancy or delivery. All children had parents with TH who did not choose to use sign language to communicate; they therefore did not have typical exposure to language, in either spoken or signed modalities, prior to receiving their CI. There were different etiologies of the hearing loss: for 23 children the cause was genetic and for 22 the cause was unknown; however, 19 children did not have results of genetic testing at the time of the study. All children had received their first CI before or at 27 months of age (*M* = 12.91 months, *SD* = 4.79 months). At the time of the study, 29 children were implanted bilaterally and 16 unilaterally (13 children had their CI in the right ear and three in the left ear). A total of 43 children had received a hearing aid (HA) before they received their first CI (age range: 3–14 months, *M* = 5.62, *SD* = 2.53). At the time of the study, 9 out of 16 children with unilateral implants were using both a CI and an HA.

Control group (TH group): 94 children with typical levels of hearing and no reported diagnosis of neurodevelopmental disorders (age range: 3.25–7.25 years, *M* = 4.48; SD = 1.12; 3-year-old: *n* = 22; 4-year-old: *n* = 27; 5-year-old: *n* = 26; 6-year-old: *n* = 16; 7-year-old: *n* = 3; boys: *n* = 52; girls: *n* = 42).

Descriptive statistics are presented in Table 1.

**Table 1:** Descriptive statistics for TH and CI groups, including variables connected with the implantation available at the time of the study

Variable	*M*	*SD*	Range
TH group (*n* = 94)			
Age (years)	4.48	1.12	3.25–7.25
CI group (*n* = 45)			
Age (years)	5.38	1.15	3.25–8.25
Age at first HA (months) (*n* = 43)^a^	5.62	2.53	3–14
Time since first HA (months) (*n* = 43)^a^	64.12	13.83	29–90
Age at CI (months)	12.91	4.79	6–27
Duration of CI use (months)	55.44	14.06	24–85
Age at second CI (months) (*n* = 29)^b^	45.24	13.92	16–66
Time between first and second CI (months) (*n* = 29)^b^	33.59	13.94	9–54
Time with both CIs (months) (*n* = 29)^b^	24.52	17.87	8–61
AAST – Q (dBHL)	33.98	6.11	23.80–48.80
AAST – N (dBHL)	–10.19	2.77	(−15.30)–(−2.50)

*Note*. “HA” = “Hearing Amplification”, “CI” = “Cochlear Implant”, “AAST-Q” = “The Adaptive Auditory Speech Test in quiet”, “AAST-N” = “The Adaptive Auditory Speech Test in noise”, “dBHL” = “Decibels Hearing Level”.

^a^Reflects a subgroup of participants who had HA before receiving CI.

^b^Reflects a subgroup of participants who had two CIs at the time of the study.

This broad age range was advantageous for this study because it allowed us to match groups closely in terms of the ages when developmental changes in ToM development are most likely to occur. Based on previous studies, we expected to find delays in ToM development in children with CIs, therefore we attempted to investigate how prolonged this delay is. Hence, the inclusion of younger children with TH (3-year-old) enabled us to identify whether older children with CIs match the children with TH in levels of ToM understanding or, alternatively, whether their FB performance might lie somewhere between that of 3-year-olds and children aged 4–5. Consequently, investigating older children with CIs enables us to identify when they are likely to acquire FB understanding.

### Procedures

A standard session lasted approximately 45 min and took place at a clinic (CI group) in a standard audiology booth for hearing testing or in kindergartens in a separate speech and language therapist’s room (TH group). It was conducted by four qualified psychologists experienced in working with children with CIs. Each participant was tested once. The assessment of sentence comprehension and ToM tasks was carried out in random order. In the CI group, the Adaptive Auditory Speech Test (AAST) was conducted by a qualified teacher of the DHH during a separate appointment. It lasted 5–7 min. Prior to the testing session, the external and internal part of the CI system was checked by the clinical engineer. Hearing sensitivity was verified within the free field audiometry.

#### ToM task

The results of previous studies often emphasized that classic FBT might be challenging for younger children due to its complexity and executive function demands (for example, Bloom & German, 2000; Rubio-Fernández & Geurts, 2013). Therefore, our task was modified in order to reduce executive load and linguistic complexity. The scenario was presented in the form of short cartoons, without introducing an elaborate storyline. The ToM task was displayed on a touch-screen laptop and participants selected the answer by touching one of the boxes. It was therefore fully automated, thereby limiting the experimenter’s impact on the child’s performance. Furthermore, no verbal answer was required from the children. Moreover, only one protagonist was introduced in the scenario—the toy changed its location of its own accord, rather than being intentionally moved by a second character. As a result, participants did not need to switch between the perspective of various characters while simultaneously inhibiting their own perspective. This ToM task was previously used with a sample of children with typical levels of hearing and has been demonstrated to be valid for the assessment of ToM development (Wysocka et al., 2020).

Each cartoon clip depicts a character (a boy or girl) who observes a toy being placed in a box. In the False Belief condition, the character leaves the scene at this point and is absent when the toy moves to another box. The character subsequently comes back to the room and the pre-recorded test question is played: “Where does the child think the toy is?”. In this condition, the subject’s and character’s beliefs about the toy’s location are incongruent. The True Belief condition is analogous to the False Belief condition and differs only in terms of the character’s presence during the relocation phase: the character leaves the room only for a short period of time and can see the toy move to the new location. Therefore, in this condition, the subject’s and character’s beliefs are congruent. The test question remains the same. Additionally, there is a No Belief condition, as a control, where the character is replaced with a color-matched geometrical shape. The test question is: “In which box is the toy?”. All conditions are matched in terms of total duration (35 s), amount of movement, and color scheme. The cartoon clips are presented in random order four times in each condition: one set of ToM stimuli contains 12 clips. Therefore, each participant could score 0–4 points on the False Belief, True Belief, and No Belief conditions.

The ToM task was preceded by a short training (described in detail in Wysocka et al., 2020) in order to familiarize the children with the experimental situation and to ensure that they understood the task and the two prerecorded questions.

#### Assessment of sentence comprehension

The “Sentence comprehension” subtest from the Language Development Test was used as a measure of the participants’ ability to comprehend the meaning of sentences with different levels of grammatical complexity (Smoczyńska et al., 2015). The Language Development Test is a standardized measure of various language skills for children aged from 4.0 to 8.11 years. Separate normalized scores are provided for each subtest, therefore they can be administered and analyzed separately. The “Sentence comprehension” subtest consists of 32 cards, each of which depicts four illustrations. It is based on the Test for Reception of Grammar: Version 2 (Bishop, 2003; Smoczyńska et al., 2015). Participants are shown the cards one at a time and are instructed to point at the picture which best illustrates the grammatically complex sentence read by the experimenter; their accuracy is measured. Participants can obtain a maximum of 32 points—one for each sentence. Here, raw scores were used for statistical analysis since some of the participants were younger than 4 years of age and their results could not be converted into standard scores.

#### The Adaptive Auditory Speech Test

The AAST assesses Speech Recognition Threshold (SRT) in noise and quiet and is designed for children aged 3 to 4 years old or older. The procedure of the test is automatic, adaptive, and only depends minimally on the participant’s vocabulary (Fels et al., 2009). The AAST is presented in an interactive form: participants listen to recorded words and have to identify the corresponding picture from a set of six (e.g., a girl, a box). In the Polish version, all items are trisyllabic words which are familiar to children. After a correct response, stimulus intensity is reduced by one step; after an incorrect response, it is increased by two steps. The step size is 5 dB for speech in quiet and 2 dB for speech in noise (Coninx et al., 2007). After seven incorrect answers, the algorithm automatically stops the procedure and calculates a threshold value based on the last six responses (Coninx et al., 2007). The range of the scores are between 10–65 dB (for quiet) and −18–4 dB SNR (for noise). The scores are reversed, therefore a greater score means worse performance. The AAST is easy to administer, with an average testing time of 2 min per condition (Fels et al., 2009). The AAST was conducted in a sound-proof booth with a loudspeaker in front of the participant.

#### Data analysis

All demographic and clinical variables were subjected to statistical analysis. Due to differences in group sizes and lack of normal distribution of the tested variables (investigated with the Shapiro–Wilk test and tests of skewness and kurtosis), between-group comparisons of the demographic variables, ToM tasks (number of correct answers calculated separately for False, True, and No Belief conditions), and level of sentence comprehension (from the Language Development Test) were performed with a series of Wilcoxon rank-sum tests (Mann–Whitney U tests). The significance level was set at *p* < 0.05. R software was used for this part of the analysis.

In order to corroborate the findings from the classical hypothesis testing, when a *p* value was between 0.05 and 0.1, FB data were further analyzed within a Bayesian framework (Bayes factor, BF_10_) using JASP (JASP Team, 2017). This enabled us to determine whether the data favor the null hypothesis over the alternative hypothesis. Bayes factors are reported below; by convention, BF_01_ < 1/3 and BF_01_ > 3 are taken as evidence in favor of the alternative and null hypotheses, respectively, while values within these boundaries are taken as providing no evidence one way or the other (Quintana & Williams, 2018).

## Results

To prepare the data, only children who completed the FBT and answered at least two of the four control questions correctly on the False Belief Test (No Belief condition; NB) were included in further analyses (N = 134; CI group: *n* = 40; TH group: *n* = 94). This step allowed us to control for the possible confounding influence of the language and memory demands of the task. Three children from the CI group quit the FBT (two 4-year-olds and a 3-year-old) and two children did not pass the control task (i.e., answered none or one of the questions correctly on the NB condition; a 3-year-old and a 4-year-old). In the TH group, all children completed the FBT and obtained a minimum of 2 points on the NB condition. Further analysis was conducted in four steps.

First, we attempted to identify how much (if at all) ToM is delayed in children with CIs. In contrast to other studies on ToM development in children with CIs, which typically include children with a wide range of ages, the aim of this study was to directly compare performance on ToM tasks at the transition age for acquiring ToM (which is typically 4–5 years). Therefore, we divided the participants into two groups: a younger group (4–5 years) and an older group (≥6 years). There were no age differences between the younger children with CIs and younger TH groups or between the older CI users and older TH groups. Four- and five-year-olds with CIs had significantly lower results on the FB condition than their peers with TH. This disparity between groups was no longer observable between the older children, as there were no differences between 6-, 7-, and 8-year-olds with CIs and their peers with TH in results on the FB condition. However, subsequent Bayesian analysis conducted on the results on the FB condition with older children resulted in a BF_01_ = 1.4, thereby providing anecdotal evidence in favor of the null hypothesis. Further analysis showed that 4- and 5-year-olds with CIs were not significantly different from 3-year-old children with TH in results on the FB condition. There were no significant differences between younger children with CIs and younger children with TH in results on either the TB or NB conditions. Similarly, there were no significant differences between older children with CIs and TH in results on the TB or NB conditions.

There were significant differences between younger children with CIs and younger children with TH in the level of sentence comprehension. Four- and five-year-olds with CIs had lower sentence comprehension than their peers with TH. These differences were still present in the older group, as children with CIs performed worse than their peers with TH on the sentence comprehension task.

Descriptive statistics and test variables for younger and older groups are presented in Tables 2 and 3, respectively.

**Table 2:** Descriptive statistics and test variables for younger TH group and younger CI group (4–5 years)

Variables	Younger TH group (*n* = 53)			Younger CI group (*n* = 16)			*W*	*p*	*r*
	*M*	*SD*	*Mdn*	*M*	*SD*	*Mdn*			
Age (years)	4.49	0.50	4.0	4.56	0.51	5.0	454.5	.622	–.059
FBT									
FB	2.09	1.67	3.0	0.88	1.41	0.0	260.0	.015	–.292
TB	3.00	1.47	4.0	3.31	1.08	4.0	465.5	.516	–.078
NB	3.81	0.39	4.0	3.69	0.60	4.0	393.0	.534	–.075
Sentence comprehension^a^	25.11	4.73	25.0	18.38	4.21	18.0	127.0	<.001	–.509
*Stanine scores* ^a^	*6.43*	*1.58*	*6.0*	*4.06*	*1.18*	*4.0*			

*Note.* Comparison of group scores by age, false belief test results and sentence comprehension with the Wilcoxon rank-sum tests. “FBT” = “False Belief Test”, “FB” = “False Belief condition”, “TB” = “True Belief condition”, “NB” = “No Belief condition”. The values represent the raw scores.

^a^Analyses for the “Sentence comprehension” subtest were performed on the raw scores. Descriptive statistics for the standard scores (stanines) are provided in italics.

**Table 3:** Descriptive statistics and test variables for older TH group and older CI group (6–8 years)

Variables	Older TH group (*n* = 19)			Older CI group (*n* = 24)^a^			*W*	*p*	*r*
	*M*	*SD*	*Mdn*	*M*	*SD*	*Mdn*			
Age (years)	6.16	0.37	6.0	6.29	0.55	6.0	25.5	.447	–.116
FBT									
FB	3.00	1.20	3.0	2.08	1.69	2.5	163.5	.102	–.249
TB	3.53	0.70	4.0	2.67	1.74	4.0	183.5	.227	–.184
NB	3.89	0.32	4.0	3.83	0.38	4.0	214.0	.582	–.084
Sentence Comprehension^b^	28.89	3.33	30.0	25.39	5.94	28.0	127.0	.021	–.357
*Stanine scores* ^b^	*6.05*	*1.72*	*6.0*	*4.52*	*2.04*	*5.0*			

*Note.* Comparison of group scores by age, false belief test results and sentence comprehension with the Wilcoxon rank-sum tests. “FBT” = “False Belief Test”, “FB” = “False Belief condition”, “TB” = “True Belief condition”, “NB” = “No Belief condition”.

^a^Group comparisons for sentence comprehension were performed for 23 children from the CI group, because one child did not complete the task.

^b^Analyses for the “Sentence comprehension” subtest were performed on the raw scores. Descriptive statistics for the standard scores (stanines) are provided in italics.

Additionally, the raw scores from the “Sentence comprehension” subtest from the Language Development Test were converted into standard scores according to the standard nine (stanine) method for participants older than 4 years of age. In this sample, approximately 19% of the younger children with CIs (4–5 years old) had stanine scores below 4 and 81% had stanine scores between 4 and 6 (*M* = 4.06; *SD* = 1.18). Concerning older children with CIs (6–8 years old), approximately 26% had stanine scores below 4 and 61% had stanine scores between 4 and 6, and 13% obtained scores above the sixth stanine (*M* = 4.52; *SD* = 2.04).

Next, we investigated the relationship between age at implantation, ToM, and sentence comprehension. After performing a median split based on the age at implantation (*Mdn* = 12 months), as in Sundqvist et al. (2014), the group with CIs was divided into two subgroups: 22 children who received their CI ≤ 12 months of age (implantation age range: 6–12 months, *M* = 9.36, *SD* = 2.04) and 18 who were implanted ≥13 months of age (implantation age range: 13–27 months, *M* = 16.89, *SD* = 4.35). There were no age differences between the children who received their implant earlier (age range: 4.42–7.75) and later (age range: 4.17–8.25 years). We then compared the children’s performance on the FBT and sentence comprehension tasks. There were no significant differences between groups on any of the conditions: FB, TB, or NB. Differences between groups on the sentence comprehension task were not significant, however the results were on the level of statistical tendency (*p* = .057), potentially suggesting that children who received their CI earlier exhibited higher sentence comprehension than the later CI group. This result is also somehow reflected in the standard scores, as approximately 5% of children (i.e., one child) from the earlier CI group had stanine scores below 4 and 90% had stanine scores between 4 and 6, while 5% obtained a score above the sixth stanine (*M* = 4.86; *SD* = 1.42). In the later CI group, approximately 44% of children had stanine scores below 4 and 44% had stanine scores between 4 and 6, while 11% obtained scores above the sixth stanine (*M* = 3.72; *SD* = 1.90).

Descriptive statistics and test variables for both groups are presented in Table 4.

**Table 4:** Descriptive statistics and test variables for earlier CI group implanted ≤12 months of age (earlier CI group) and CI group implanted ≥13 months of age (later CI group)

Variables	Earlier CI group^a^ (*n* = 22)			Later CI group^b^ (*n* = 18)			*W*	*p*	*r*
	*M*	*SD*	*Mdn*	*M*	*SD*	*Mdn*			
Age (years)	5.64	0.85	6.0	5.56	1.20	6.0	205.0	.852	–.030
FBT									
FB	1.59	1.59	1.5	1.61	1.82	0.5	199.5	.977	–.005
TB	3.14	1.49	4.0	2.67	1.57	3.0	240.0	.208	–.199
NB	3.86	0.35	4.0	3.67	0.59	4.0	227.5	.257	–.179
Sentence comprehension^c^	24.38	5.65	26.0	20.33	6.46	2.5	257.0	.057	–.305
*Stanine scores* ^c^	*4.86*	*1.42*	*5.0*	*3.72*	*1.90*	*4.0*			

*Note.* Comparison of group scores by age, false belief test results and sentence comprehension with the Wilcoxon rank-sum tests. “FBT” = “False Belief Test”, “FB” = “False Belief condition”, “TB” = “True Belief condition”, “NB” = “No Belief condition”.

^a^Reflects children with CI implanted from 6 to 12 months of age. Group comparisons for sentence comprehension were performed for 21 children from the earlier CI group, because one child did not complete the task.

^b^Reflects children with CI implanted from 13 to 27 months of age.

^c^Analyses for the “Sentence comprehension” subtest were performed on the raw scores. Descriptive statistics for the standard scores (stanines) are provided in italics.

In the third step, we conducted a series of zero-order and partial non-parametric Spearman correlations on the group with CIs to test the relationships of duration of CI use with FBU, sentence comprehension, and age at implantation. The child’s age was controlled for in every correlation, because it was significantly related to FBU (*r_s_* = 0.42, *n* = 40, *p* = 0.007) as well as sentence comprehension (*r_s_* = 0.66, *n* = 39, *p* < 0.001). Because there were significant correlations between the duration of CI use and the child’s age (*r_s_* = 0.88, *n* = 40, *p* < 0.001), we did not report correlations when controlling for the duration of CI use.

Analysis revealed a significant positive relationship between FBU and sentence comprehension (*r_s_* = 0.51, *n* = 39, *p* = 0.001), which was still significant after controlling for the child’s age in months (*r_s_* = 0.36, *n* = 39, *p* = 0.025). Surprisingly, there was no significant correlation between age at implantation and FBU (*r_s_* = −0.03, *n* = 40, *p* = 0.860), which remained nonsignificant after controlling for the child’s age (*p* = 0.777). There was no significant relationship between the age at implantation and sentence comprehension (*p* = 0.211). However, after controlling for the child’s age, a significant negative relationship emerged between the level of sentence comprehension and age at implantation (*r_s_* = −0.36, *n* = 39, *p* = 0.029). The level of sentence comprehension was higher when a child had received their CI at a younger age.

There were significant positive correlations between the duration of CI use and FBU (*r_s_* = 0.39, *n* = 40, *p* = 0.014) and level of sentence comprehension (*r_s_* = 0.72, *n* = 39, *p* < 0.001). The correlation between the duration of CI use and the level of sentence comprehension remained significant after controlling for the child’s age (*r_s_* = 0.41, *n* = 39, *p* = 0.011). However, the correlation between the duration of CI use and FBU was no longer significant after controlling for age (*p* = 0.801).

Lastly, we examined the relationship between FBU, sentence comprehension, and AAST results. There were significant correlations between FBU and AAST in noise (*r_s_* = −0.38, *n* = 39, *p* = 0.016) and quiet (*r_s_* = −0.36, *n* = 40, *p* = 0.022). However, these relationships were no longer significant after controlling for the child’s age (*p* > 0.05). A similar pattern was found with the child’s level of sentence comprehension as a dependent variable. There was a significant correlation between sentence comprehension and AAST result in noise (*r_s_* = −0.47, *n* = 38, *p* = 0.003) but not in quiet (*p* = 0.375). However, after controlling for the child’s age, both relationships were not significant (*p* > 0.05).

Finally, we investigated whether it is possible for children with CIs pass FBTs at the typical age (4–5 years old). We assumed that a child passed the FBT when he or she gave at least three correct answers out of four. We identified two children who displayed typical ToM development (a girl and a boy). Both children were 4 years old at the time of the study. Both had sentence comprehension results within the range typical of their peers with TH. One child had received their implant at the age of 7 months and one at the age of 13 months. Both were implanted bilaterally and used a hearing aid before implantation (one child started using a HA at the age of 4 months and the other one at the age of 5 months).

## Discussion

As far as the authors are aware, this is the first study to explore FB understanding in a group of children who were DoH who had all received their first implant between 6 and 27 months of age. In agreement with previous investigations (Ketelaar et al., 2012; Sundqvist et al., 2014), the present study found that ToM performance in children who were DoH was delayed relative to the expected level of their peers with TH. We observed this discrepancy in children aged 4–5. This is an important result, as there is a consensus that the transition from failure to success on the FBT takes place around this age.

The results for children aged 6–8 were equivocal. Using classical statistical tests, we found no significant differences between the older CI and TH groups in terms of ability to infer the mental states of others. However, based on Bayesian analysis, we cannot be confident that the null hypothesis is true. Furthermore, the data also did not support the alternative hypothesis. This does not agree with previous data, which reported a significant delay in ToM acquisition (Ketelaar et al., 2012; Peterson, 2004; Remmel & Peters, 2009). For example, Peterson (2004) reported that children with CIs or HAs displayed a delay in ToM performance of about 3–5 years compared to typically developing children. This inconsistency might be due to (at least) two reasons. First, in contrast to other studies (see Sundqvist et al., 2014), we only recruited children who received their first implant between 6 and 27 months of age, with mean age at implantation = 12.9 months. As hearing loss may act as a barrier to social communication for children who are DoH (Peterson, 2020; Sundqvist et al., 2014), earlier cochlear implantation may buffer against greater ToM delay, as suggested by our results.

Secondly, we used a modified FBT which minimizes demands on language and working memory. We did this in response to the criticism of classic paradigms for their excessive complexity and executive function demands (Baillargeon et al., 2010; Devine & Hughes, 2014; Rubio-Fernández & Geurts, 2013; Wellman et al., 2001). However, the majority of other studies on children with CIs used traditional ToM tasks, that is to say, short narratives presented with spoken or signed language and accompanied by illustrations (e.g., see Ziv et al., 2013). The high linguistic demands of these tasks could have affected ToM performance in DoH children with delayed language development due to prelingual deafness. In our study, linguistic demands were minimized and children heard only two types of questions: “Where does the child think the toy is?” and “In which box is the toy?”

Despite this simplified version of the ToM task, children with CIs aged 4–5 (the younger group), but not older children, performed significantly worse than their peers with typical hearing. This could be possibly explained by poorer sentence comprehension, as demonstrated by our results. According to the meta-analysis conducted by Milligan et al. (2007), comprehension of syntactically complex sentences is significantly related to FBU. This ability could support the representation and tracking of the mental state of the character, which is essential in the false belief test (Astington & Jenkins, 1999). Studies with children who are DoH, including those with CIs, have also indicated a relationship between grammar comprehension and FBU (e.g., de Villiers & de Villiers, 2012; Peters et al., 2009). However, the older group with CIs (aged 6–8) still demonstrated poorer sentence comprehension than their peers with typical hearing, despite the fact that performance on the FBT did not differ between these groups. This result suggests that there might be additional sources of ToM delay in this population, other than poorer sentence comprehension.

A growing body of data supports the hypothesis that the emergence of belief attribution is influenced by conversational–communicative input (Meristo et al., 2016; Wellman & Peterson, 2013). Parental attribution of mental states to their young infants (e.g., “You think mommy’s funny?”) provides infants a vehicle for integrating the thoughts, feelings, and actions of themselves and others. In line with this, it has been demonstrated that the frequency of mothers’ mental state talk is associated with their children’s use of such language (Furrow et al., 1992; Hughes & Dunn, 1998) and children’s later understanding of the mind (Meins et al., 2003). The complexity of a mother’s speech is concordant with the language skills of their children and parents of typically developing children increase their use of mental state terms as their children get older (Bartsch & Wellman, 1995; Dunn et al., 1991). This line of research has clearly shown that parental mental state talk in everyday conversation facilitates the acquisition of mental state concepts by their children (Slaughter & Peterson, 2012). Therefore, the positive effect of “mindful” conversations and interventions which might enhance ToM development in children should be made well-known by specialists to parents and caregivers. Some initial work has already been done in this regard. For example, Wellman and Peterson (2013) showed that training based on thought bubbles improved ToM abilities in DoH children. Other studies found that ToM can be scaffolded with explicit instruction (Stanzione & Schick, 2014), writing (Chilton et al., 2019a, 2019b) or through using fiction books to explore the topics of thoughts and feelings (Chilton & Beazley, 2018). However, this issue was not tested directly in our study as we did not control for the quantity and quality of caregiver talk directed at children.

Our results showed no significant difference in FBT performance between children implanted before the first year of life and those who received their implant between 13 and 27 months of age. There was also a lack of correlation between FBT performance and either age at implantation or duration of CI use (after controlling for the children’s age). These results were contrary to our expectations but probably reflect the fact that only 1 month separated the groups. For example, Sundqvist et al. (2014) demonstrated that children who received CIs earlier perform better on FBTs than their peers who receive their CIs later. However, these results cannot be directly compared with ours because the studies differ substantially in age criteria for early and later implantation. Sundqvist et al. (2014) defined early implantation as “before 27 months of age”. In our study, all children received their first CI before 27 months of age (in fact, all but one were implanted before 2 years of age). Therefore, although it is indisputable that earlier implantation may lead to a more typical trajectory of ToM development in DoH (due to its positive effect on spoken language development), based on our results we cannot posit that children who received CIs during their first year of life will develop ToM at a more age-typical rate than children implanted between the first and second years of life.

However, we observed a tendency for poorer sentence comprehension in the later CI group and correlation between this variable and age at implantation as well as the duration of CI use, which might suggest that earlier implantation facilitates spoken language development. This is in line with studies that show that the optimal time for cochlear implantation is before the second year of life, when the central auditory pathways are maximally plastic to sound stimulation, resulting in typical acquisition of oral language (Kral & Sharma, 2012).

We did not observe any relationships between FBU, sentence comprehension, and quality of speech perception (measured with AAST; after controlling for children’s age). This result is not surprising due to the fact that AAST assessment revealed that the majority of children had only slight problems with speech perception. High quality of speech perception means that a child can receive spoken language input (including mental state talk) incidentally, usually by overhearing conversations, even in challenging (noisy) listening situations. This is important as incidental learning via better access to spoken language has been reported to promote the development of ToM (Wiefferink et al., 2013) and pragmatic skills (Boons et al., 2013).

The present study showed that some subsets of children with CIs are able to pass FBTs within the typical age range (4–5 years old). As only two children aged 4–5 who succeeded on the FBT were identified, we cannot draw conclusions based on this data. However, it is worth mentioning that both of them received their first CI around the age of 1-year-old and they performed equally well as their peers with TH on the sentence comprehension task. It is possible that earlier implantation enables such children to successfully develop the language skills essential for success on FBTs. This result is also in line with a number of studies that show great variability in outcomes following implantation (for example, Zgoda et al., 2019). More studies are needed to explore the factors that influence the development of ToM after implantation.

There are several limitations to this study. Firstly, we did not control for the family’s socioeconomic status (SES) or the children’s general intellectual functioning. Family SES could explain part of the variance in performance on the FBT, as has been indicated by previous research (Devine & Hughes, 2018). Also, general intellectual abilities are usually controlled for in studies examining ToM. However, all DoH children were recruited by a qualified teacher of DHH children who enrolled only children without concurrent disabilities and within the range of typical psychomotor development. Secondly, we did not include any measure of parental mental state talk which, as discussed above, could have a significant impact on a child’s later ToM development (Devine & Hughes, 2018; Peterson, 2020). Lastly, we were not able to successfully test a group of 3-year-olds with CIs, as the recruited children did not yet have sufficient level of language competency to understand the instructions for the FBT.

Another important note is that ToM cannot be seen as a unitary construct and success on explicit FBTs is not equivalent to having a fully mature understanding of other minds. False belief task performance is just a single step along a long trajectory of increasingly sophisticated abilities that enable the understanding of emotional dissembling, non-literal speech like sarcasm and irony, and social faux pas (Beaudoin et al., 2020; Dvash & Shamay-Tsoory, 2014; Shamay-Tsoory et al., 2010).

In subsequent work we will address these limitations. Furthermore, we hope to extend our sample to younger children with CIs.

## Conclusions

Taken together, our findings indicated that the development of FBU was delayed until the age of 6 years in most of children with CIs who started to use a CI before and around their second birthday, even when the task had minimal linguistic demands. However, this developmental delay seems to be less profound than other studies have reported, probably due to earlier implantation resulting in greater availability of effective linguistic input. Children with CIs “catch up” in ToM development with their peers with TH when they have had the benefit of their implant for a longer period. At the age of testing, ToM skills were equally developed in children who received their first CI before the age of 1 year and between their first and second birthday.

Our study also has clinical significance. The key message is that parents and those who work with DoH children with CIs must recognize the importance of social cognition in addition to the typical focus on language outcomes. Caregivers should be made aware that children who are DoH experience challenges with ToM as a result of the potentially restricted language environment prior to implantation. Therefore, postimplantation interventions should include the assessment of ToM and caregivers should be given information about how to support their children in building their ToM skills.

## Fundings

This work was supported by the National Science Centre [2017/25/B/HS6/01624].
